# Misdiagnosis and Treatment of Corneal Complications Caused by Suture Exposure After Buried-Suture Double-Eyelid Blepharoplasty

**DOI:** 10.1007/s00266-024-04045-7

**Published:** 2024-05-06

**Authors:** Xin Jin, Hong Zhang

**Affiliations:** Key Laboratory of Basic and Clinical Research of Heilongjiang Province, Eye Hospital, The First Affiliated Hospital of Harbin Medical University, No. 23 Youzheng Road, Harbin, 150001 Heilongjiang Province People’s Republic of China

*Level of Evidence IV* This journal requires that authors assign a level of evidence to each article. For a full description of these Evidence-Based Medicine ratings, please refer to the Table of Contents or the online Instructions to Authors www.springer.com/00266.

Dear Editor,

Thanks to Professor Zuguang Hua and Peng Wei for their interest and careful reading of our study. We appreciate the opportunity to respond to this letter to the editor.

The purpose of this article is to remind ophthalmologists that if patients with corneal ulcers have undergone buried-suture double-eyelid blepharoplasty, ophthalmologists should turn the upper eyelid in time to find suture exposure factors, so as to avoid misdiagnosis.

In our article [1], we have made a brief summary of the advantages and disadvantages of different sutures, it is also reminded that if you go to a regular hospital, you can properly avoid suture exposure caused by unskilled surgical operation. 6-0 or 7-0 translucent monofilament and multiple nylon suture were used for buried-suture double-eyelid blepharoplasty [2–6]. However, multi-center clinical trials are still needed to explore which type of suture is more suitable for buried-suture double-eyelid blepharoplasty. Thanks again to Professor Zuguang Hua and Peng Wei for his recognition and valuable comments on our article.
